# Psychotic Cannabis Withdrawal: A Clinical Case

**DOI:** 10.7759/cureus.31465

**Published:** 2022-11-13

**Authors:** Berta Ramos, Ana Filipa Santos Martins, Eva Sofia Lima Osório

**Affiliations:** 1 Psychiatry, Centro Hospitalar Universitário de São João, Oporto, PRT

**Keywords:** sleep deprivation, dependence, psychosis, withdrawal, cannabis

## Abstract

Cannabis use has been associated with several psychiatric comorbidities and there appears to be a dose-response relationship between the intensity and duration of its use and the risk of psychosis. More commonly, acute episodes of cannabis induced-psychosis manifest immediately following exposure, are precipitated after the use of large amounts of cannabis, resolve with abstinence, and are of shorter duration than those observed with primary psychotic disorders.

Cannabis withdrawal symptoms usually manifest when heavy, prolonged consumption of this substance is interrupted or significantly reduced. The withdrawal syndrome may include sympathetic autonomic hyperactivity, irritability, anxiety, sleep disturbance, and reduced appetite. On the other hand, cases of psychosis induced by cannabis withdrawal are rare.

In this case, we present a 32-year-old healthy woman without personal or family psychiatric history who showed a heavy and continued consumption of cannabis since she was 10 years old, without developing any psychiatric symptoms. However, recently she experienced two brief psychotic episodes with disorganized behavior and persecutory delusions, both episodes happening a week after discontinuing cannabis consumption.

## Introduction

Cannabis use has been associated with several psychiatric comorbidities, including mood, anxiety, and psychotic disorders, particularly in the case of consumption at an early age [1]. While these associations are well established, the mechanisms involved are not fully understood, with inconsistent and even contradictory findings [2].

Cannabis withdrawal syndrome occurs when heavy and prolonged consumption of this substance is interrupted or significantly reduced. Its symptoms usually appear within 24 to 72 hours and last for about one to two weeks [3]. It can mimic withdrawal syndromes from other substances, such as alcohol or opioids, with sympathetic autonomic hyperactivity, stomach pain, tremors, sweating, fever, headaches, irritability, anxiety, sleep disturbance, and, more rarely, psychosis [4-6].

In addition to the cannabis withdrawal syndrome, the Eleventh Revision of the International Classification of Diseases (ICD-11) includes cannabis-induced psychotic disorder, which is characterized by “psychotic symptoms that develop during or soon after intoxication with or withdrawal from cannabis” and that “are not better explained by a primary disorder” [7]. While intoxication-induced psychotic cases are commonly described on literature, psychotic cases after cannabis cessation are rarely reported.

## Case presentation

We present the case of a 32-year-old woman, with an acute psychotic episode admitted to the psychiatry emergency department, where she was brought by the police for disorganized behavior. The only psychiatric record she presented was a visit to the emergency service with a similar episode to the current one, which happened four years before. However, at that time she did not follow the recommendations given after discharge, such as attending a follow-up consultation and taking the prescribed antipsychotic medication.

In the emergency department, she was agitated, restless, and aggressive and she stated that she was being threatened by someone she did not know, who was following her and wanted to kill her and her family. She also believed she had a bomb inside her body, which she tried to remove by scratching herself in the neck region, and that her ovaries had been disturbed by an exterior force. In this first evaluation, the patient could not tell when she started to feel like this.

She was disheveled, with poor hygiene, barefoot, and wearing wet clothes. She was unpleasant and uncooperative in the psychiatric interview, but attentive and aware of the time, current location, and situation. Mood and affect were extremely irritable. She displayed disorganized speech and persecutory delusions, although she did not show any sign of hallucinations. The insight and judgment were poor, and she did not want medical help or medication. She had no history of any medical condition, seizure activity, or head trauma. There were no symptoms consistent with mania or hypomania.

The neuroimaging (head computerized tomography) and analytic study (including hemogram, leucogram, ionogram, thyroid, renal and hepatic function analysis, syphilis, borrelia, HIV, HCV, and HBV serologies, inflammatory markers, alcohol seric levels) were normal. The urine toxicology screen taken was positive for cannabis and negative for cocaine, opiates, and psychostimulant drugs.

During hospitalization, it was possible to understand that she presented altered behavior and initial and sleep maintenance insomnia for two weeks. These symptoms started two days after she stopped using cannabis (she was consuming 2 g per day in the last 15 years). A week previous coming to the emergency department, she was more agitated, with disorganized behavior, and with total insomnia. Because of that, she was no longer able to practice her job as a hairdresser and engage in activities with her social network as she used to.

Four years back, she presented a psychotic episode similar to the current one, also after the suspension of cannabis use. After being evaluated in the emergency department, she was discharged home and diagnosed with unspecified inorganic psychosis. At that time, she was medicated with an oral antipsychotic and oriented to a psychiatry consultation, which she missed. After that, she maintained the medication for one week, was abstinent for three months, and returned to her daily routines and functionality. After resuming cannabis use, she denied having new symptoms. A family history of psychosis or other severe mental illness was not present.

Regarding cannabis use, she began using cannabis at the age of 10, half a joint a day. At 12 years old, she was smoking 10 joints a day, having reduced to two joints a day when she started working at the age of 17. Shortly after, she increased the drug use to six joints a day, about two grams of cannabis, which she has maintained until the present. She stated that consumption helped her to relax and interact with others. Moreover, she was an active smoker (10 cigarettes a day) and did not present any other substance use. On the psychiatric ward, she was treated with aripiprazole 15 mg (once a day) and diazepam 5 mg (twice a day). After the appropriate pharmacological intervention, she showed gradual improvement over one week of hospitalization.

On the day of discharge, she did not present psychotic symptoms, namely delusions or hallucinations. She was prescribed the same medication on discharge and referred to psychiatric consultation for follow-up. She was evaluated in ambulatory consultation, where she admitted resuming smoking cannabis again, at a lower dose (about 0.5 g per day), while taking the medication prescribed. She presented psychopathological stability, without psychotic symptomatology. She was doing a hairdressing course and has resumed her social life, apparently regaining her former functionality.

## Discussion

This clinical case describes a psychotic episode that appears to have occurred due to the abrupt cessation of cannabis use. The patient was using cannabis daily for the last 24 years and with regular use of 2 g for the last 15 years.

Cannabis use is a well-recognized risk factor for psychotic symptoms and there appears to be a dose-response relationship between the intensity and duration of its use and the risk of psychosis [8,9]. According to European Drug Report 2020, around 1% of adults in the European Union countries are daily or almost daily (20 days or more in a month) cannabis users and the majority of these are under 35 years old [10].

Usually, the use of cannabis is associated with acute episodes of psychosis that manifest immediately following exposure, which is typically precipitated after the use of large amounts of cannabis, resolve with abstinence, and are of shorter duration than those observed with primary psychotic disorders [11,12], though the user has also been associated with episodes of psychosis persisting beyond the period of intoxication [13].

Cannabis withdrawal is dependent on the amount of consumption pre-cessation, gender, genetic and environmental factors. Besides the physical signs, the psychiatric symptoms that have been described in the literature include irritability, anger or aggression; nervousness or anxiety; sleep difficulty; decreased appetite; restlessness; depressed mood, and rarely, psychosis. However, psychiatric manifestations are still an infrequent and misunderstood phenomenon [4-6].

Both withdrawal symptoms (particularly sleep deprivation) and a brief psychotic episode were observed in this young adult patient, who presented to the emergency psychiatry department with persecutory delusions. The timeline of this patient’s episodes strongly suggests psychosis induced by cannabis withdrawal, whose symptoms are consistent with previous reports available in the literature [13]. We also considered that sleep deprivation, consequence of cannabis withdrawal, contributed to the onset and evolution of psychotic symptomatology in this case.

## Conclusions

Although uncommon, it is necessary to raise awareness of cannabis withdrawal psychosis to better clarify the mechanisms of cannabis abstinence as a psychotic factor. Additionally, this case highlights the need to provide more attentive care to withdrawal symptoms, particularly when promoting drug abstinence.
